# Observations on the long-lived Mossbauer effects of ^93m^Nb

**DOI:** 10.1038/srep36144

**Published:** 2016-11-08

**Authors:** Yao Cheng, Shi-Hui Yang, Michael Lan, Chih-Hao Lee

**Affiliations:** 1 Department of Engineering Physics, Tsinghua University, Beijing, Haidian, 100084, China; 2 Haidian, 100085, Beijing, China; 3 Ho Kang Technology Co., Ltd., Hsinchu, 30091, Taiwan; 4 Department of Engineering and System Science, National Tsing Hua University, Hsinchu, 30013, Taiwan; 5 Institue of Nuclear Engineering and Science, National Tsing Hua University, Hsinchu, 30013, Taiwan

## Abstract

Several observations of the Nb long-lived Mossbauer phenomena are presented, in consequence of an irradiation increased by an order of magnitude compared with previous work. These are 1) two β decays of ^182^Ta and ^92m^Nb are enhanced, i.e., ^182^Ta is now 200 times faster than in previous results while ^92m^Nb is twice as fast as normal; 2) γs emitted from two β decays compete to eject electrons in a winner-takes-all rule, rather than by superposition; 3) abrupt spectral changes reveal three decay phases of ^182^Ta; 4) the biphoton γγ of ^93m^Nb is released from the sample for the first time; 5) the γγ distribution is narrow, in contrast to the broad γγ spectrum obtained from independent nuclei; 6) Nb K-lines super-radiate along the long sample axis; 7) collective scattering of multipolar MeV γs. The manipulation of nuclear decay speeds demonstrated here highlights a potential application of this work in cleaning up the nuclear wastes.

To explain the resonant absorption of long-lived Mossbauer effects mediated by the biphoton γγ^1^,^2^,^3^, a theory has been developed to interpret the variety of available experimental observations. A brief introduction to the physics of this theory and its extension to 4 entangled γs is required. Results of ^93m^Nb experiments presented now reconfirm the large number of ^103m^Rh observations made before 2008^1^,^3^, which are briefly summarized in the Supplementary information.

In the multipolar nuclear transition of long-lived Mossbauer nuclides^4^, simultaneous γγ occur in opposite directions to preserve the γ energy by eliminating their recoil energy. Once resonant absorption occurs between two identical nuclei in the same neighbourhood, the probability of spontaneous γ emission yields a superradiant factor of 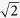
5. The spontaneous γγ emission thus becomes 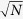-fold effective in a crystal containing N identical nuclei in resonance^6^.

Delocalised γγ absorb N phonons to acquire a mass, in the form of the so-called nuclear exciton^1^, having an emerging magnetic quantum number of m = 0^7^. The exciton spreads in a region containing N/M identical nuclei in a micron range with M (∼10^12^) excitons and N (∼10^22^) nuclei in a crystal.

The AC Stark effect produces Rabi sidebands around the characteristic γs, regardless of atomic or nuclear transitions^1^,^3^. Mollow triplets^3^ were observed for Rh K-lines and ^103m^Rh γ at 39.8 keV. The vacuum Rabi oscillation Ω_R_ for M/N ≪ 1/2 is so fast that the recoil phonon never leaves, leading to an entanglement between γγ and the phonon^2^. The superradiance has an Avogadro number of N rather than N^1/2^, resulting from the superposition of N nuclei × N phonons in resonance^1^. A detailed evaluation provided in the Supplementary information reveals the ^103m^Rh superradiance ∼10^28^ ≫ N. The old model of ^103m^Rh given in the previous report^1^ is therefore incomplete.

Neither ^93m^Nb γγ nor its Rabi splitting has previously been observed^1^,^2^, and till now the exact value of Ω_R_ has not been known. Nevertheless, Ω_R_ must be greater than 30 meV in order to survive the thermal agitation at room temperature. Assuming that the M4 Γ_γγ_/Γ_γ_ branching ratio is mainly attributed to the low-lying E2 Raman transition as well as the nuclear size^8^,^9^, we fine-tuned the value of the ^137^Ba 11/2^−^ excited state for ^93m^Nb to 10^−6^. Four factors, i.e., the 30-meV Ω_R_, the 16-year half-life, the internal conversion, and the Γ_γγ_/Γ_γ_ branching ratio, give a γγ superradiance ∼10^33^. The rotational symmetry of ^93m^Nb is spontaneously broken by the absorption of N nuclear magnons^10^.

Applying the new observation of Ω_R_∼100 eV, the superradiance becomes ≫ N^3/2^ and the above-mentioned ^93m^Nb model is again incomplete. Bosonic excitons prefer antiferromagnetic ordering to lower the energy. There are two magnon polarizations in the antiferromagnetic ordering. The M4 exciton of ^93m^Nb may consist of a pair of γγs that acquire one more degree of freedom to absorb the residual magnon polarization. The exciton chains align themselves in the direction of the long sample axis. The photon flux therefore depends on the sample geometry. The emerging magnetic quanta of pairing 4 γs give rise to a multiplet rather than a simple triplet. The central energy of the pairing γγs is one quarter of the transition energy, which is 7.71 keV for ^93m^Nb with E_0_ = 30.82 keV^4^.

## Results

**Figure 1:**
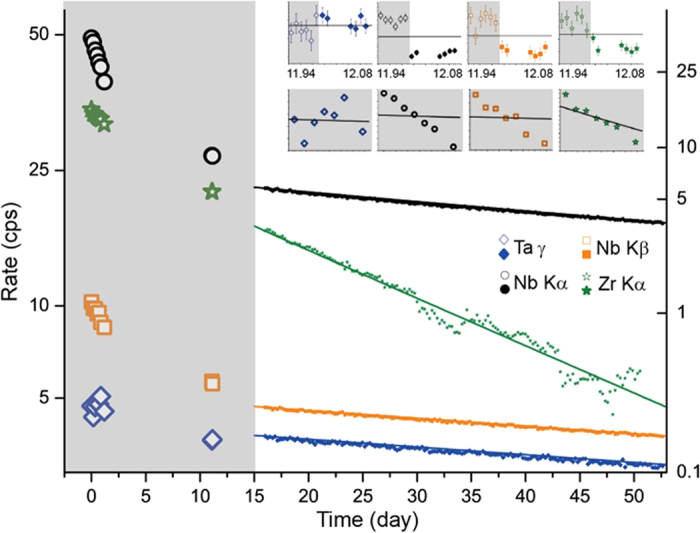
Decays of four x-rays in counts per second (cps) against their reference lines of half-lives listed below. The left-hand axis shows the rates of Nb Kα (black dot, 111.4 d), Nb Kβ (orange square, 111.4 d), Ta γ (blue diamond, 111.4 d), and the right-hand axis shows the rates of Zr Kα (green star, 10.15 d). Removing its contamination makes Zr Kα very sensitive to the temperature shift of the spectra. Data points of Zr Kα after day 51 are deleted because of their overly large fluctuation. The hollow symbols show data points measured at the incline position highlighted by the gray background, while the solid symbols show data points measured at the contact position. The data points taken at the incline position are multiplied by a factor of calibration using the detected total counts (see Fig. S1 in the Supplementary information). The insets show the calibration between two measuring positions, where the K x-rays of Nb and Zr are anisotropic. The details of the first 7 data points and their reference lines are shown in the insets blow.

Figure 1 shows the decays of four x-rays, where Ta γ @ 67.8 keV represents the β decay of ^182^Ta and Zr Kα represents the electron capture + β^+^ decay of ^92m^Nb. X-rays have two characteristic time constants corresponding to ^92m^Nb and ^182^Ta. The contributions of the slow ^93m^Nb (half-life of 16 years) and ^94^Nb (half-life of 2 × 10^4^ years) are negligible. The varying time constants are demonstrated by the initial 9 data points of the four decays shown in Fig. 1, which were taken in the same position during the period of hazardous radioactivity.

**Figure 2:**
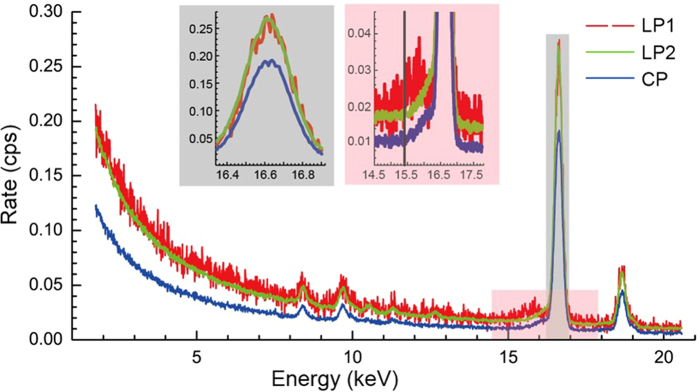
Superradiance of Nb K-lines along the long sample axis. Two spectra of LP1 and LP2 were taken in the longitudinal position and one spectrum of CP was taken in the contact position at day 116. LP1 was taken during the transition of ^182^Ta decay from phase 1 to phase 2. One inset gives the detailed superradiance of Nb Kα. The Nb Kα/Ta γ ratio is ∼6 for the contact position. The Nb Kα/Ta γ ratio is ∼8 for the longitudinal position, which equates to >8, because the Pb wrapping foils block Nb K-lines but do not block Ta γ. The other inset shows the broadened γγ peak just at the moment, at which the spectral change took place. The vertical black line highlights the γγ energy of 15.4 keV. Another peak is probably located @ 15.9 keV (see Fig. S3 and Fig. S4 in the Supplementary information). Therefore, a conservative estimate gives the γγ FWHM of ∼600 eV, which then gives a true γγ FWHM of ∼400 eV.

The initial sample position with inclination was replaced by a contact position at day 12. The calibration between the two positions using the same activated sample (Fig. 1) demonstrates an interesting anisotropy at about 20%. More anisotropic x-rays are shown in the Supplementary information. We conclude that the main fluxes of the Nb K-lines and the Zr K-lines do not run along the 1-mm axis of the sample. Instead, x-rays super-radiate along the 3-cm axis, where the Nb Kα increased by more than 40% (see Fig. 2). It is worth noting that the anisotropic impurity channel of Ni revealed by the sub-Poissonian photon statistics^2^ is not caused by the phenomenon discussed here.

**Figure 3:**
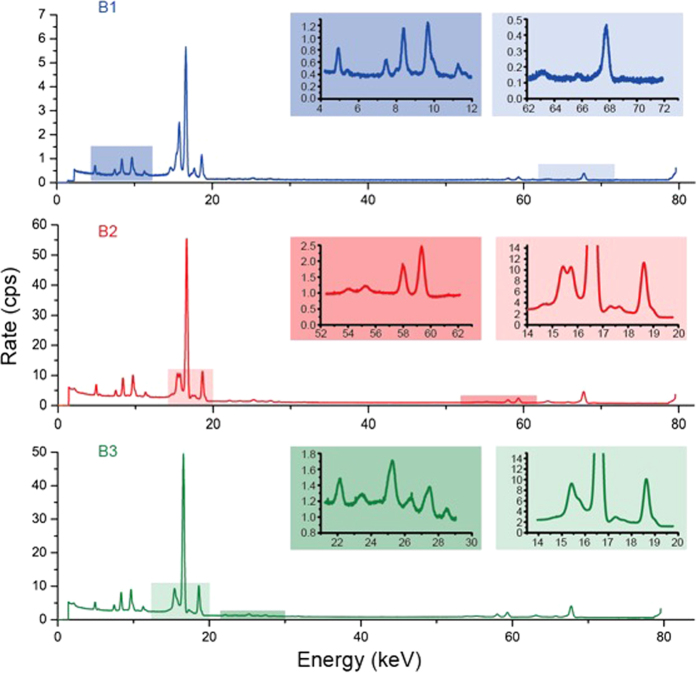
Spectral evolutions. Line B1: accumulation of the first 7 data points; line B2: accumulation of the first 80 data points; line B3: accumulation of the next 80 data points. Major x-rays are identified, i.e., V Kα @5.0, V Kβ @5.4, Quad @7.5, W Lα @8.4, W Lβ @9.7, W Lγ @11.3, biphoton γγ @15.4, Zr Kα @15.7, Nb Kα @16.6, Zr Kβ @17.7, Nb Kβ @18.6, Hf Kα_2_ @54.6, Hf Kα_1_ @55.8, W Kα_2_ @58.0, W Kα_1_ @59.3, Hf Kβ_3_ @63.0, Hf Kβ_1_ @63.2, Hf Kβ_2_ @65.0, Ta γ @65.7, W Kβ_3_ @67.0, W Kβ_1_ @67.2, Ta γ @67.8, W Kβ_2_ @69.1 in keV. It should be noted that the measured energy of Hf Kα is 400 eV lower than normal. Several peaks in the energy ranging from 22 to 29 keV are not identified but have frequently been observed before^1^,^2^. These peaks disappear after one year and are probably due to radioactive impurities.

The first 7 data points of Ta γ shown in Fig. 1 depict a chaotic decay of ^182^Ta, which is, however, at least three times faster than the normal half-life of 114.43 days. This enhanced decay then relaxes within 10 days. Taken together, the 160 data points give a 3% enhanced half-life of 111.4 ± 0.3 d. The Hf K-lines (see Fig. 3 and Fig. S2 in the Supplementary information) appear to follow the ^182^Ta decay. An α branching channel to reach a stable ^178^Hf from ^182^Ta is considered.

With some care we inspected the high-energy γs using an HPGe detector. Only the characteristic γs emitted from the ^182^Ta and the ^94^Nb decays were found (see Figures S6 and S7 in the Supplementary information). The readings of two γs @ 706.2 keV and @ 871.1 keV reveal the major excitation of ^94^Nb in the sample, the density of which was ∼1000 times higher than the ^182^Ta density at day one. Neither the MeV α particle nor the characteristic γs of ^178^Hf or ^178^Lu were found. The MeV α particles escaping from the sample were probably too rare to be caught by the α-particle detector used. Figure 2 shows that the Hf K-lines disappeared after day 116, when the Zr Kα from ^92m^Nb decayed to 0.03 counts per second (cps). We observed the same transition at 0.02-cps Zr Kα using the same detector from the sample of a similar size in 2012^1^. At around the same time, the ratio of W Kα/Ta γ increased from 0.70 to 3.9 after day 116.

The ^92m^Nb decay is characterized by the Zr Kα in Fig. 1. Again, the first 7 data points reveal a doubled decay speed, which recovered to the normal half-life of 10.15 d within 10 days. ^92m^Nb is an isomeric γ excitation of *J*^*π*^ = 2^+^. The 135-keV M5 transition to the ^92^Nb ground state of *J*^*π*^ = 7^+^ is negligible with respect to the major β decay channel^4^. The Nb K-lines with a half-life of 4.2 days strictly follow the ^92m^Nb decay.

**Figure 4:**
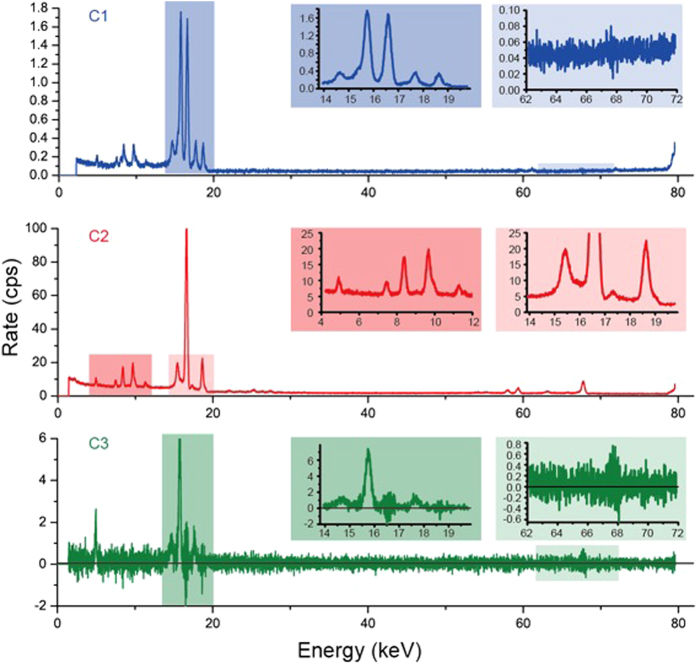
Accumulation of the decomposed spectra. Line C1 obtained using differential mapping between lines B1 and B2 + B3 under the condition of cancelling Ta γ @ 67.8 keV; line C2 is the spectrum *Ta(i*) decomposed using eq. 1 with a half-life of 111.4 d; line C3 is the spectrum *Zr(i*) decomposed using eq. 1 with a half-life of 10.15 d. C2 contains x-rays following the ^182^Ta decay. C3 contains x-rays following the ^92m^Nb decay. Note that the left Rabi sideband of Nb Kα shows up at right hand side of the non-Gaussian γγ peak @15.4 keV of C2 line. Two figures C1 and C3 are very different, i.e., C1 contains Nb x-rays and the biphoton γγ, while C3 does not.

Figure 3 shows the spectral evolution in two periods. We decomposed the 160 spectra in the contact position using two characteristic half-lives (111.4 and 10.15 d), as shown in Fig. 4 using the equation


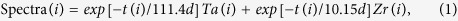


where Spectra(*i*) are the count rates in the 8192 channels, Ta(*i*) are x-rays contributed by the ^182^Ta decay, Zr(*i*) are x-rays contributed by the ^92m^Nb decay of, *i* is the index of 160 data points, and t(*i*) is the time of measurement. No x-rays strictly depend on ^92m^Nb, except the Zr K-lines and a peak @ 14.7 keV (see Fig. 4). We failed to identify this γ @ 14.7 keV to be the left Rabi sideband of Zr Kα, because its right Rabi sideband is invisible beneath the Nb Kα peak. Another small peak @ 17.5 keV follows the ^182^Ta decay, which may be the right Rabi sideband of Nb Kα. Its left counterpart @ 15.9 keV is difficult to identify in Fig. S3 and S4 of the Supplementary information. The two sidebands disappeared together after day 116 (see Fig. 2). No pronounced spectral migration of Nb K-lines given in previous work^2^ was found in these 160 data points.

V Kα (4.95 keV) and V Kβ (5.43 keV) with a branching ratio of 10:1 are identified to be the impurity channel of V, which has a similar time-varying time constant to that observed for the Ni impurity channel in previous work^2^ (see Fig. S2 in the Supplementary information).

Two pronounced peaks, i.e., γγ @ 15.4 keV (E_0_/2) and Quad @ 7.5 keV, emerge in Fig. 4, the time constant of which strictly matches the ^182^Ta decay, except for the first 7 data points. The Quad peak is apparently not the Ni Kα in previous work^2^, because the corresponding Ni Kβ is entirely absent. Furthermore, the broadband x-rays spread over the entire spectrum also follow the ^182^Ta decay. Two peaks of γγ and Quad disappeared with the Hf K-lines together after day 116 (see Fig. 2), when the low-energy broad-band x-rays between 2 and 5 keV abruptly increased by a factor of more than three, from 9 cps to 30 cps.

**Figure 5:**
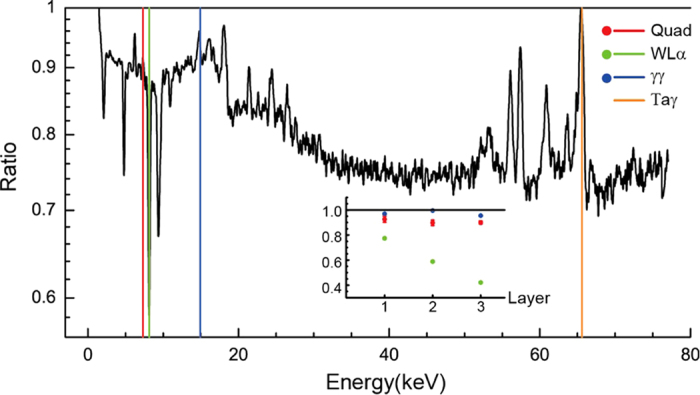
Coincident arrival of multiple γs at the detector. The spectral ratio between no-layer and 3-layer Al foils is obtained by normalizing the Ta γ @ 67.8 keV and an average of Gaussian with a sigma of 10 channels. The energy positions of the Quad, the W Lα, the γγ, and the Ta γ are highlighted by the vertical lines. The inset shows the development of the highlighted peaks by inserting Al foil layer by layer, the numbers of which are given in Table 1. Note the Quad count @ 7.5 keV deceased much less than the W Lα count, while the γγ increased on insertion of Al foils. Decreasing broadband x-rays all over the spectral range reveals they are coincident arrivals of multiple γs. We failed to identify a peak located @ 6.39 keV.

The low-energy broadband x-rays remained unchanged on insertion of a 0.1-mm Pb foil after day 116 (see Fig. S5 in the Supplementary information), while they are reduced overall following insertion of a thin Al foil before day 116 (see Fig. 5). The exponential shape seen in Fig. S5 (not a Compton plateau) reveals the random arrivals of a short-burst γs isotopically emitted into the 4π solid angle, which are probably generated by multipolar γs after day 116. The short burst lasted less than 120 ns. Multiple γs arriving at the detector are thus treated as a single count by the multi-channel analyzer, which reports a 100% live-time. The 70% live-time before day 116 reveals a long burst of a γ train, which is probably generated by the speculated α particle.

We inserted the Al filter layer by layer just one week before the spontaneous spectral change at day 116. The photo-electric attenuations of one 25-μm Al foil are roughly 30% @ 7.5 keV and 6% @ 15 keV, respectively. Figure 5 and Table 1 show the results obtained using increasing numbers of Al layers. The photo-electric attenuations of γγ and Quad are obviously abnormal. We conclude that these represent entangled γs emitted from ^93m^Nb.

The ratio of Nb Kα/Ta γ in Fig. 2 increased from 6 to 8 by rotating the sample to the longitudinal position, while the broadband x-rays between 2 and 5 keV increased by 50%. The 15.4-keV γγ was just disappearing at the moment of rotation of the sample, and it disappeared altogether on rotation back to the contact position half an hour later. The FWHM of γγ was ∼600 eV (see Fig. 2).

We applied three multipolar MeV γs from ^137^Cs and ^60^Co, i.e., 662 keV (M4), 1173 keV (E2), and 1333 keV (E2), to demonstrate the collective scattering of nuclear exciton in Table 2. The collective scattering strongly depended on the multipolarities of impinging γs. The dead time of detector in Table 2 reelected the total incoming counts. The impinging E2 γs increased 10% of the internal γ @ 1121 keV from the ^182^Ta decay, while only increased 1% of the total counts. The M4 γ did not change the total counts and the internal γ counts too much. Instead, more M4 γ was scattered away by the nuclear exciton. The M4 γ @ 662 keV from ^137^Cs is also a long-lived Mossbauer γ with a half-life of 2.55 minutes, the first-order thermal Doppler shift of which vanishes^11^. We estimate its coherent length ∼100 m using the second-order Doppler shift. In contrast, the coherent lengths of E2 γs from ^60^Co are on the μm order, as restricted by the first-order Doppler shift.

## Discussion

Although no atomic transition emits x-rays >120 keV, we observe broadband x-rays over a wide range of energies beyond 200 keV, the distribution of which contains no Compton plateau (see Fig. 2). These broadband x-rays followed the slow ^93m^Nb decay in the old single-crystal sample^2^, when ^182^Ta vanished in 2015. ^93m^Nb cannot provide x-rays >30 keV, so we must have a coincident arrival of multiple γs, as revealed by inserting a filter (see Fig. 5 and Fig. S5 in the Supplementary information). These facts reveal the presence of a high-energy source in the sample, which we then identify as ^94^Nb.

One ^94^Nb decay gave roughly one ^92m^Nb decay probably via a Raman branching at day one. Two E2 γs from ^94^Nb scatter the ^92m^Nb to the virtual 4^+^ or 6^+^ states, which return to the ^92^Nb ground state shortly. A similar Raman scheme doesn’t apply for ^93m^Nb, because there is no available 6^−^ state of ^92^Nb^4^. It requires an extreme Raman cross-section to double the ^92m^Nb decay speed. The following discussions are an attempt to shed some light on these matters.

This enhanced Raman scattering should not spontaneously stop within 10 days, unless the multipolar γs emitted from the ^92m^Nb decay or the ^92m^Nb excitations themselves cooperated with each other. The E2 γs @ 0.93 MeV emitted from the ^92m^Nb decay has a near-resonant coupling with the E2 transition @ 0.95 MeV of ^93^Nb. Coherent ^93^Nb nuclei in an exciton provide a collective scattering, which is amplified by a factor of ∼10^10^ giving rise to a strong exciton-field coupling with a Ω_R_ ≫ 1 MeV^5^,^6^. MeV E2 γs are therefore stored in a small “cavity” of the μm-size exciton. Note that E2 γs in Table 2 are from an external source. The internal E2 γ sources shall coherently move with the surrounding nuclei, that tremendously enhance the coupling strength. The photonic reservoir may thus provide a spontaneous cooperation to terminate the accelerated ^92m^Nb decay. This strong coupling also applies to the E2 γs emitted from ^94^Nb, which induce broadband x-rays following the ^93m^Nb decay. Annihilation of one ^93m^Nb exciton releases the storing E2 γs, which create a short burst of isotropic γs.

We have observed three phases of the ^182^Ta decay in the past ten years. Now they are 1) appearance of Hf K-lines with the 3% branching before day 116; 2) disappearance of Hf K-lines triggered by the vanishing ^92m^Nb after day 116; 3) appearance of Ta K-lines with 1% branching^2^ in the coming year.

Note that the parity of dilute ^92m^Nb dictates ^182^Ta that is 1000 times denser. The M4 γs from ^93m^Nb probably changes the ^182^Ta parity (*J*^*π*^ = 3^−^) by Raman accelerating the β decay in phase 2, i.e., via the 1^+ 182^Ta state @ 593 keV to the 2^+ 182^W state @100 keV, as revealed by the ratios between 100 keV/113 keV in two phases (Figures S6 and S7 in the Supplementary information). No other branching channel has yet been found.

6 MeV is required to emit a neutron from the ^182^Ta, which appears to be a pathological conclusion made in the previous report^2^. Although the α branching of ^182^Ta releases energy, the same pathology remains. Roughly 4 MeV is required to make the 3% α tunnelling observable^4^. We speculate that several MeV E2 γs from ^94^Nb stored in a photonic reservoir cause the α tunnelling out of ^182^Ta in phase 1 and the photo-neutron in phase 3.

No γγ @ 15.4 keV and Quad @ 7.5 keV were seen previously^2^ and γγ now disappear together with the Hf K-lines. We therefore suggest that the α particle creates an umklapp phonon to release γγ and Quad.

The ^92m^Nb decay dominated the day-one Nb x-rays. Later on, these are contributed wholly by the ^182^Ta decay. Two β decays were in competition with each other, and even their decay rates at day 16 and their β stopping powers were similar^4^. The vanishing ^92m^Nb contribution of the 160 data points reveals weak β activity in ejecting Nb K electrons. Instead, we consider the enhanced Raman scattering by multipolar γs.

The ratio of Nb Kα/Ta γ was 4.5 in the presence of Ta K-lines^2^ but 6 before and after day 116. It increases to more than 8 if the sample is rotated through 90 degrees (Fig. 2). One ^92m^Nb decay created one Nb K x-ray at day one, while one ^182^Ta decay created more than two Nb K x-rays. Multipolar γs emitted from ^182^Ta exhibit multiple inelastic scatterings featured by the macroscopic cross-sections.

Taking two independent radioactivities together produces a superposition of their spectra. The winner-takes-all interference between two different β decays reveals the spontaneous cooperation of multipolar γs emitted within nanoseconds, several cps of which hardly co-exist at all according to conventional wisdom. High-energy multipolar γs are easy to be stored by the near-resonant ^93^Nb. In contrast, the low-energy spin-1 x-rays are difficult to be hold by the ^93^Nb nuclei. The spin-1 Nb K-lines along the long sample axis also reveals the same result that the superradiance is established among emitters of the same kind. The 100-ps propagation time of Nb K-lines is too short to give the co-existing K-lines in sample. The sample must contain a photonic reservoir to maintain the traces of all superradiant γs.

The Quad peak deviates from the 7.71-keV 4γs by 200 eV. Further investigation is required to understand whether the energy reductions of Quad and Hf Kα (Fig. 3) are actually two sides of the same coin. If the exciton contains 4 γs rather than just 2 γs, the long-observed puzzle of the missing Ta L-lines^2^ is then resolved. The Nb standing wave between two nearest neighbouring Nb atoms (2.86 Å) is 4.34 keV (see the ^103m^Rh results in the Supplementary information). Two defect modes locate @ 9.87 + δ and @ 5.54-δ keV. Assuming δ∼0.2 keV, the 10-keV γ is not enough to eject electrons on the L1 and L2 shells of Ta, while the rapid Rabi oscillations inhibit the photoelectric ejection of the Ta L3 electrons with an ionization energy of 9.881 keV.

The energy distribution of an individual γγ is usually very broad^4^, while a narrow width shows up the non-Gaussian FWHM of 130 eV and 170 eV at the contact and incline positions, respectively (see Fig. S3 in the Supplementary information). The γγ width further increased to 400 eV at the longitudinal position (Fig. 2). According to our theory, the γγ width will be the 0.03-eV Debye energy. The γγ peak is thus a quintuplet mainly contributed by a Ω_R_∼100 eV of m =± 1 at the contact position. The pronounced broadening of the γγ FWHM in the longitudinal position reveals the anisotropic γγ of m = ± 2 with a Ω_R_∼200 eV. The speculated 800-eV Rabi sidebands around Nb Kα in Fig. S3 is probably driven by 4 γs with a 200-eV Ω_R_, which disappear most likely due to the parity change after day 116.

## Conclusions

The well-known photonic physics as promoted by the AMO society has been extended to the realm of nuclear physics in this report. We suggest that multipolar γs from ^94^Nb stored in a photonic reservoir accelerate two different β decays. ^182^Ta β decay abruptly switches its branching channels, as dictated by the parity of diluted ^92m^Nb. Multipolar γs emitted from two different β decays compete with each other, rather than superposing together to eject the Nb K electron. Their Raman cross-sections are extremely large. The superradiance along the long sample axis reveals a coherent enhancement of Nb K-lines, which have no intrinsic coherence at all. The Nb K-lines cannot leave the coherent trace-records in the sample, unless a photonic reservoir exists. Taking these observations together leads us to a long-predicted coherence for all ^93m^Nb excitation in the crystal^1^. The superradiance of 10^36^ ≫ N^3/2^ gives rise to the reported 4γ model.

## Methods

In a set of experiments, we newly activated four niobium polycrystalline sheets of 99.99% purity (3 cm × 1.5 cm × 1 mm). We refer to sample No. 3 in this work. The method is as detailed in the reference 1, except that the irradiation time was two hours without the Cd shielding. The ^92m^Nb density was thus increased by a factor of 20 compared with the previous result. We used the same silicon detectors located at Hsinchu in the previous report^1^, which are the Si-PIN type (XR-100CR) manufactured by AMPTEK in Bedford, MA 01730, USA. The day-one measurement started at 2016/2/4.

There are 3 different measuring positions, i.e., 1) an incline position before day 12: the sample is 1-cm away with a 30-degree angle of inclination; 2) a contact position after day 12: the sample is directly in contact with the beryllium window of the detector; and 3) a longitudinal position: the 3-cm long sample axis is parallel to the normal vector of the beryllium window. The sample area fully covers the 0.5-inch ϕ beryllium widow of detector at the contact position. Three samples (No. 1, No. 2, and No. 3) were wrapped by 5 layers of 0.1-mm Pb foil together, such that the Pb shielding opened an area of three surfaces (1 mm × 1.5 cm) in contact with the beryllium window in the longitudinal position.

Two kinds of filter were inserted between the sample and the beryllium widow. One was a 25-μm Al foil, the other was a 0.1-mm Pb foil. Their photo-electric attenuations were verified by the calibration sources of ^241^AM and ^55^Fe.

The live-times of detector were insensitive to the incoming rate during the calibration between incline position and contact position, i.e. 70% for 120 cps and 230 cps, respectively. However, it changed to 100% after day 116.

We cooled the sample in liquid nitrogen between day 53 and day 81. Two detectors of the same type are applied, i.e., one for the measurements before day 116 and the other for the measurements after day 116. The first detector failed to work at day 116 and it was replaced by the second detector.

The high-energy x-rays were taken by a HPGe detector (CANBERRA GC0518, Coaxial-Ge type) with ϕ 4.25 cm and a length of 4.1 cm, which was calibrated by ^137^Cs (10 μCi), ^60^Co (1 μCi) and ^241^AM (1 μCi) and located in a box of the 3-cm Pb shielding. The temperature of measuring environment was stable (27 ± 0.5 °C). We manipulated the dead time of HPGe detector to avoid any possible pile-up errors induced by the ^182^Ta decay in the samples, when 20% dead time appeared in Table 2.

## Additional Information

**How to cite this article**: Cheng, Y. *et al.* Observations on the long-lived Mossbauer effects of ^93m^Nb. *Sci. Rep.* **6**, 36144; doi: 10.1038/srep36144 (2016).

**Publisher’s note:** Springer Nature remains neutral with regard to jurisdictional claims in published maps and institutional affiliations.

## Supplementary Material

Supplementary Information

## Figures and Tables

**Table 1:** The photo-electric attenuations of six x-rays, i.e., Nb Kα, Nb Kβ, γγ, W Lα, W Lβ, and Quad, by comparing their ratios between inserting 1, 2, 3 layers of Al foil and no Al foil.

Layer/X ray	Nb Kα	Nb Kβ	γγ	W Lα	W Lβ	Quad
1	0.958 ± 0.004	0.974 ± 0.006	.970 ± 0.006	0.774 ± 0.010	0.876 ± 0.007	0.97 ± 0.02
2	0.985 ± 0.004	1.014 ± 0.005	.996 ± 0.006	0.589 ± 0.009	0.727 ± 0.007	0.90 ± 0.01
3	0.946 ± 0.002	0.990 ± 0.003	.955 ± 0.003	0.421 ± 0.005	0.579 ± 0.004	0.900 ± 0.005

The ratios of the Ta γ counts @ 67.8 keV are normalized to be unity. Note that the γγ ratios are greater than the Nb Kα ratios by 3 sigma, while the Quad ratios are greater than the W Lα ratios by 70 sigma.

**Table 2:** Collective scattering of multipolar MeV γs.

Source	Sample location	Real time	1121 keV	662 keV	1173 keV	1333 keV
No	0 cm	1262 s	336.7 ± 0.7	—	—	—
No	1 cm	1221 s	300.3 ± 0.6	—	—	—
^137^Cs	0 cm	1282 s	337.0 ± 0.7	101.5 ± 0.5	—	—
^137^Cs	1 cm	1243 s	301.0 ± 0.6	108.6 ± 0.5	—	—
^60^Co	0 cm	1274 s	369.3 ± 0.7	—	10.7 ± 0.2	15.3 ± 0.2
^60^Co	1 cm	1233 s	301.3 ± 0.5	—	11.8 ± 0.2	16.4 ± 0.2

Two sources of ^137^Cs and ^60^Co provided three major γs, i.e., 662 keV (M4), 1173 keV (E2), and 1333 keV (E2).

No significant scattering (<3%) was found by inserting a pristine sample beneath two sources. In contrast, only 90% of the MeV γs penetrated the active sample. Data in cps were taken with a live time of 1000 s in two sample locations, either beneath the source (0 cm) or near the source (1 cm). Note that 10% of the E2 γ @ 1121 keV from the ^182^Ta decay in sample was released by the ^60^Co source, while the total counts increased only 1%.
