# Supplementary material for: Observations on the long-lived Mossbauer effects of 93mNb
Source: Sci Rep. 2016 Nov 8;6:36144. doi: 10.1038/srep36144 (PMC5099924; doi:10.1038/srep36144)
Supplement: Supplementary Information [file srep36144-s1.pdf]

On-line Supplementary

Observations on the long-lived Mossbauer effects of ^{93m}Nb

Yao Cheng[1*], **Shi-Hui Yang**[2,†], **Michael Lan**[3], **Chih-Hao Lee**[4,5*]

[1]Department of Engineering Physics, Tsinghua University, Beijing, Haidian, 100084, China
[2]Haidian, 100085, Beijing, China

[3]Ho Kang Technology Co., Ltd., Hsinchu, 30091, Taiwan

[4]Department of Engineering and System Science, National Tsing Hua University, Hsinchu, 30013, Taiwan

[5]Institue of Nuclear Engineering and Science, National Tsing Hua University, Hsinchu, 30013, Taiwan

*Corresponding authors Y. Cheng (yao@tsinghua.edu.cn) and C.-H. Lee (chlee@mx.nthu.edu.tw)
†Present address: Department of Physics and Astronomy, Stony Brook University, Stony Brook, NY 11794, USA

Brief summary of the ^{103m}Rh experiments before 2008

The research into long-lived Mossbauer effects started in 2004. The multipolar Mossbauer transitions of ^{45m}Sc, ^{107m}Ag, ^{109m}Ag, and ^{103m}Rh were activated by the bremsstrahlung from a 6-MeV linac. It was soon realized that single isotope is required to give significant nuclear resonance. Most of the early studies were carried out using the E3 ^{103m}Rh with a transition energy E_0 = 39.8 keV. A brief summary[1,3] is provided here with some newly developed interpretations.

The Rabi oscillation induced by the Mossbauer resonance gives the characteristic sidebands by the AC Stark effect, regardless of whether the transitions are atomic or nuclear. The Rabi sidebands were identified around the major emissions of Rh K-lines (@20 and @23 keV) and the ^{103m}Rh γ @ 39.8 keV.

Increasing the ^{103m}Rh density beyond 3×10^{11} cm^{-3}, the ^{103m}Rh underwent a transition from regime I to regime II, which was characterized by the density-dependent Rabi splitting Ω_R and the non-linear pumping efficiency. On further pumping beyond a threshold of 10^{12} cm^{-3}, the ^{103m}Rh entered regime III, where Ω_R switched between two values every 20 minutes on a couple of occasions. The critical densities among the regimes were decreased by lowering the sample temperature revealing two phase transitions among three different states of ^{103m}Rh.

All the observed Ω_R were vacuum splitting, as revealed by ^{103m}Rh per atom << 1/2. Ω_R was 50 eV, not depending on the ^{103m}Rh density in regime I. A Mollow triplet of the 39.8-keV γ with an $\Omega_R \sim$ 400 eV in regime II was barely identified by the HPGe detector used. This open Ω_R will be resolved using a silicon drift detector in the near future.

We estimate the ^{103m}Rh superradiance to be 10^{28} >> N by four factors, i.e., the 50-eV Ω_R, the ^{103m}Rh lifetime, the internal conversion, and the $\gamma\gamma$ branching ratio (assuming $\Gamma_{\gamma\gamma}(E1+E2)/\Gamma_\gamma(E3) \sim 10^{-5}$), which reveals an incomplete $\gamma\gamma$ model as reported previously. The superradiance of the exciton must be considered.

A vacuum Ω_R shall not depend on the excitation density and temperature. To interpret the density- and temperature-dependent Ω_R in regime II, Y. Cheng suggests that excitons condensed into liquid droplets, where the rotational symmetry of nuclei was spontaneously broken. The locally enhanced pumping efficiency of the droplets exponentially increased with their growth until the phase transition into regime III was triggered. $\Omega_R >> 50$ eV in regime II was irreversible during the three-hour monitoring. The open Ω_R did not recover back to 50 eV, even when the ^{103m}Rh excitation density decayed to a level much lower than 3×10^{11} cm^{-3}. This observation supports the model of liquid droplets. The ratio between the shrinking droplets and vapour remained constant during the decay.

As soon as the rotational symmetry of ^{103m}Rh is spontaneously broken in the liquid phase, exciton absorbs N nuclear magnons leading to a non-vanishing expectation of the orientated photon flux. The superradiant factor of ^{103m}Rh $\gamma\gamma$ became $N^{3/2} \sim 10^{33}$ in the liquid phase, where the vapour-phase $\gamma\gamma$ disappeared for $\hbar\Omega_R \geq E_0$. Instead, three entangled γs on the fcc plane exhibiting a three-fold symmetry, e.g., [1, 1/2, 1/2], [1/2, 1, 1/2] and [1/2, 1/2, 1], probably replaced the $\gamma\gamma$ configuration. The $\gamma\gamma\gamma$ branching ratio of the E3 ^{103m}Rh is smaller than its $\gamma\gamma$ branching ratio by orders of magnitude, e.g., assuming 10^{-4}, such that the 10^{33} superradiance gives rise to an observed vacuum $\Omega_R > 400$ eV.

Nb is a photonic crystal for the entangled γs, the propagation of which is forbidden. Biphoton $\gamma\gamma$ with half ^{103m}Rh energy at 19.9 keV leaks from the sample boundary. The differential map between the sample orientations revealed the $\gamma\gamma$ and its two sidebands located at 17.4 keV and 22.4 keV from the contamination. This successful mapping is evidence of the broken rotational symmetry at room temperature. The superradiant Rh K-lines and 39.8-keV γ along the long sample axis was also verified by applying a magnetic field. Their photon fluxes depended on the applied field while their permeability depended on the sample orientations. The 5-keV energy between sidebands has two contributions $E_s + 2\delta$, i.e., $E_s = 4.61$ keV is the standing wave between two nearest neighbours with a distance of 2.69 Å apart and $\delta \sim 0.2$ keV is the energy shared by the neighbouring nuclei on the Bragg mirrors. This standing-wave triplet also emerged by following the insertion of a Cu filter due to the fact that the filter absorbs one of the coincident $\gamma\gamma$ count at 39.8 keV to give an extra count at 19,9 keV.

Anomalous broadband x-rays spreading over the entire spectrum up to 600 keV were verified by their decay and by inserting Cu and Ta foils. Two long-lived radioactivities of ^{195m}Pt and ^{193m}Ir were activated by the 6-MeV bremsstrahlung. There might be other high-energy sources in the sample.

Three peaks located at 17.3 keV, 18.3 keV and 19.3 keV emerged on insertion of the Cu foil or cooling with liquid nitrogen. They may be the left standing-wave sidebands featured by fcc lattice constants, eg. 1.83 keV is the 3.8 Å standing wave. Their right standing-wave sidebands were invisible beneath the broadband x-rays.

Details of the x-rays emitted from the Nb sample in this report

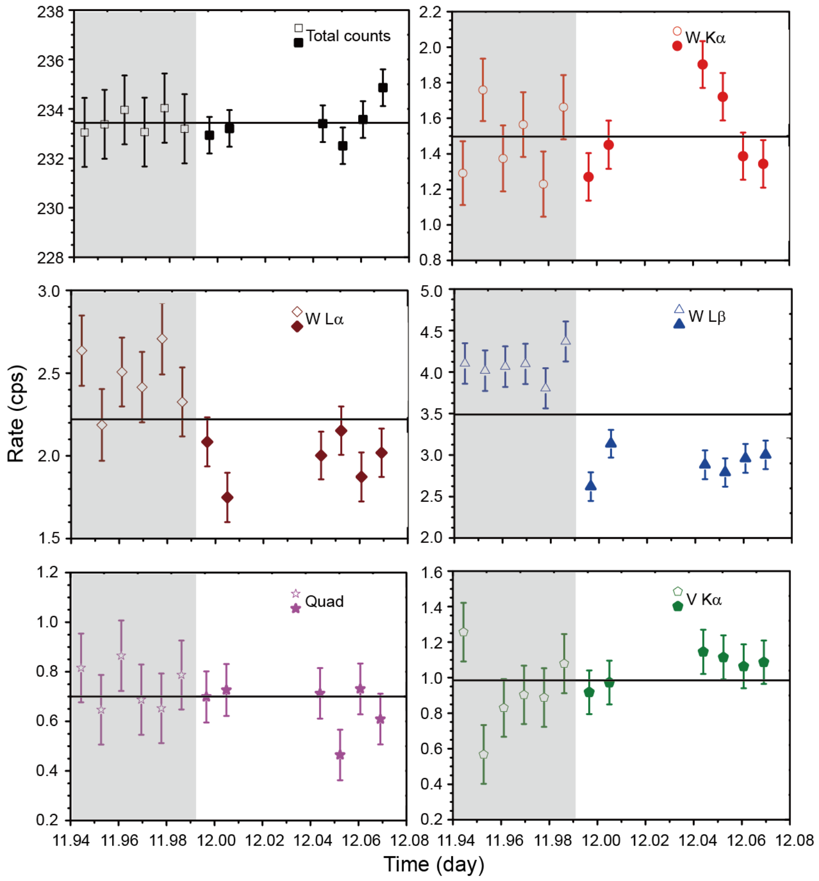

Supplementary Figure S1: Detailed calibrations between the two measuring positions, as shown in Fig. 1. The first six data points multiplied by a factor of calibration are taken at the incline position, while the last six are taken at the contact position. 1: rates of total counts, which are the numbers applied to calibrate two positions; 2: W Kα; 3: W Lα; 4: W Lβ; 5: Quad is the count rates at 7.5 keV; 6: V Kα. Two W L-lines are obviously anisotropic.

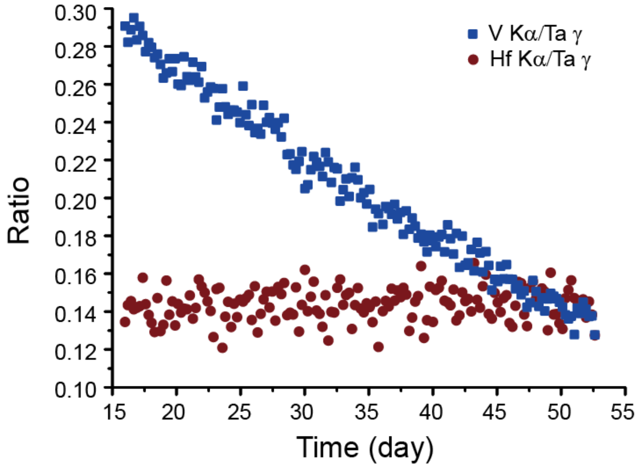

Supplementary Figure S2: Decays of the V impurity channel and Hf Kα, where the count ratios V Kα/Ta γ and Hf Kα/Ta γ are presented for the 160 data points at the contact position. This picture precludes the V Kα contribution from the β decay of ^{51}Cr with a half-life of 27.7 d.

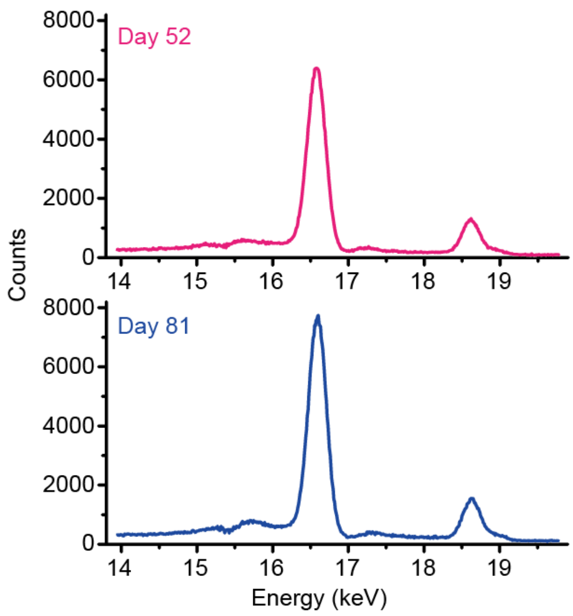

Supplementary Figure S3: Sidebands around the Nb Kα at day 52 and day 81. The contamination of Zr Kα is negligible, as demonstrated by two spectra. The manipulation of removing the $\gamma\gamma$ peak is detailed in Fig. S4 of this supplementary information. These figures reveal two issues, i.e., the sidebands are located at 15.8 keV and 17.4 keV while the $\gamma\gamma$ peak is broader than the Nb Kα peak. The shape of $\gamma\gamma$ peak is not Gaussian and its FWHM values are $\sim$ 420eV during the first period and ~380 eV during the first period, while the calibrated FWHM of the detector at 15 keV is 250 eV. The true FWHM of the $\gamma\gamma$ peak is thus 130-170 eV.

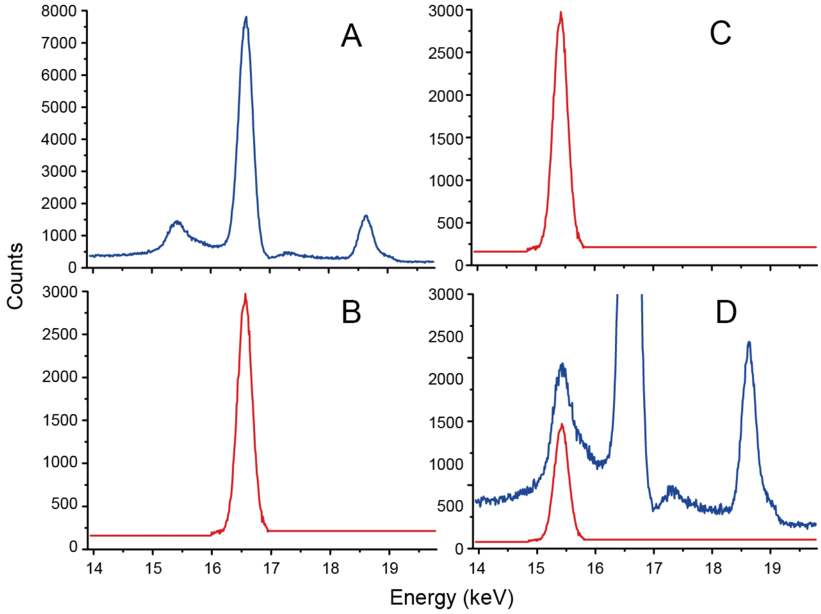

Supplementary Figure S4: Manipulation of Fig. S3 in this supplementary information. A: the original spectra at day 52 (day 81). B: the calibration using pure Nb irradiated by the 30-keV e-beam. C: the Nb Kα of the calibration is moved to the left to coincide with the $\gamma\gamma$ peak. D: the differential map is then produced by subtracting the original spectrum from the calibration curve multiplied by a factor under the condition necessary to recover the background level. The Nb Kα widths of A and B are equal, while the $\gamma\gamma$ peak is broader than the Nb Kα peaks of A and B.

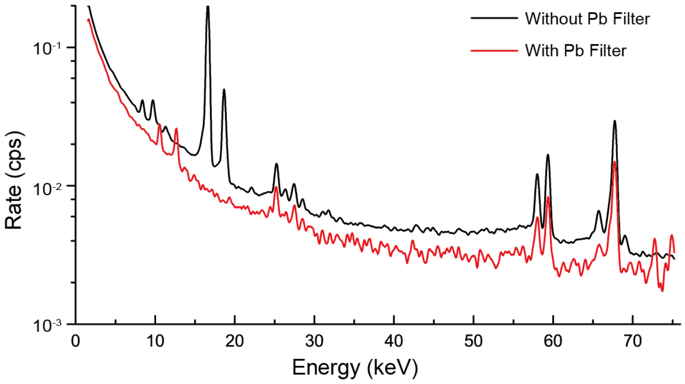

Supplementary Figure S5: Two spectra at the contact position. One is the spectrum without Pb filter. The other is the spectrum after inserting a 0.1-mm Pb foil, which entirely absorbs the Nb K-lines but not Ta γ @ 67.8 keV. The low-energy broadband x-rays show an exponential shape, as revealed by the logarithmic scale of the vertical axis. To make this presentation easier to read, the spectra have been average by a Gaussian with a sigma of 30 channels.

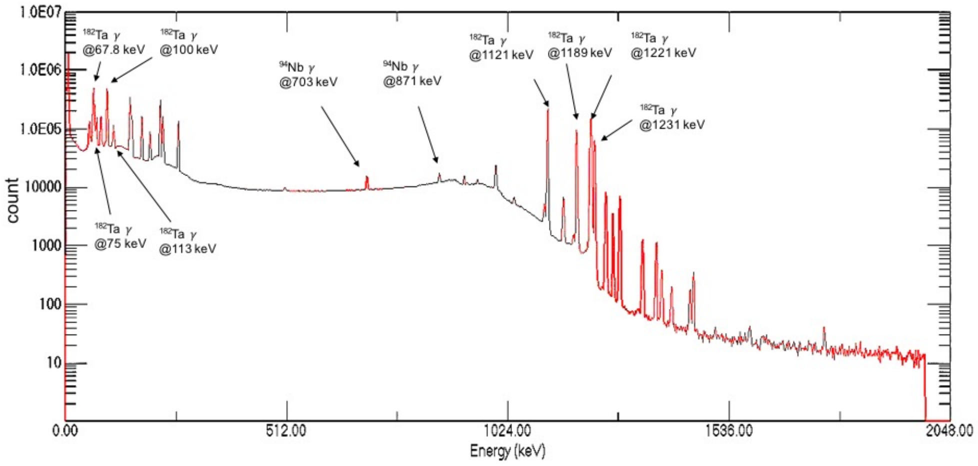

Supplementary Figure S6: Phase-1 ^{182}Ta. The ratio of 100 keV / 113 keV was 5.9. An unknown γ @ 75 keV was the coincident arrival of two γs @ 31.7 keV and @ 42.7 keV, which was absent in the spectra taken by the silicon detectors. The total count rate was 400 cps.

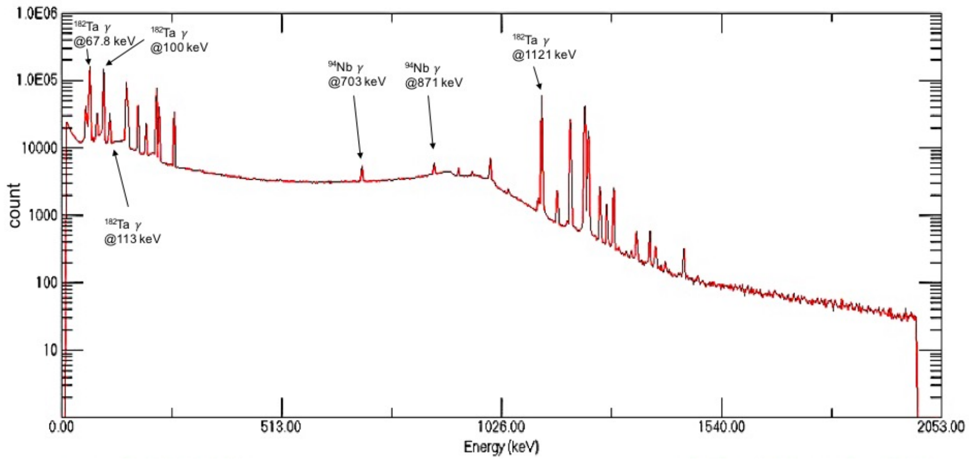

Supplementary Figure S7: Phase-2 ^{182}Ta. The ratio of 100 keV / 113 keV was 7.7. The unknown γ @ 75 keV disappeared, while the count rate was ~10^4 cps.
